# Supplementary material for: Content-rich biological network constructed by mining PubMed abstracts
Source: BMC Bioinformatics. 2004 Oct 8;5:147. doi: 10.1186/1471-2105-5-147 (PMC528731; doi:10.1186/1471-2105-5-147)
Supplement: Additional File 2 — The original results of the above study (non-essential files are deleted to keep the file size under the limit set by BMC bioinformatics). [file 1471-2105-5-147-S2.bz2 › chilibotAdditionalFile2/dip05/281ID2498083E1104/html/FOS_JUNB.html]

 


 **FOS** and **JUNB** 
  
Found 830 abstracts in PubMed, retrieved 05.  
 

 What does Google say? 
 PDF only 
| .edu only 

---

**Interactive relationship** (e.g. stimulation, inhibition, etc)

**Non-interactive relationship** (e.g. studied together, co-existance, homology, etc.)

- In contrast, mice exposed and re exposed only to the context, without foot shock, did not show behavioral signs of contextual fear conditioning and exhibited significantly less expression of c  **Fos**  and  **JunB**  in CA1 neurons.  Ref: 12882314 Genes Brain Behav, 2003
- Using specific polyclonal antisera against c  **Fos** ,  **JunB** , c Jun and JunD, we tried to identify the candidate transcription factors of the immediate early gene family which may contribute to the molecular processes during contextual memory reconsolidation.  Ref: 12882314 Genes Brain Behav, 2003
- Genes that were commonly down regulated in both PB T cells and Jurkat T cells included CDK inhibitors p16, p19, p27, proapoptotic caspases, and the transcription factors c  **fos**  and jun B  [ **JUNB** ] .  Ref: 12808112 Lab Invest, 2003
- In these mice context dependent memory retrieval evoked a marked induction of c  **Fos**  and  **JunB** , but not of c Jun and JunD, in pyramidal CA1 neurons of the dorsal hippocampus.  Ref: 12882314 Genes Brain Behav, 2003
- Supershift electrophoretic mobility shift assay indicated that  **JunB** , JunD, and c  **Fos**  components of AP 1 were particularly affected.  Ref: 12857751 J Biol ChemJ Biol Chem, 2003
- Memory retrieval after contextual fear conditioning induces c  **Fos**  and  **JunB**  expression in CA1 hippocampus.  Ref: 12882314 Genes Brain Behav, 2003
- Acute injection of morphine induces expression of the immediate early genes c  **Fos**  and  **JunB**  in several forebrain regions of the rat, in part through an N methyl D aspartate NMDA receptor dependent mechanism.  Ref: 12809699 Neuroscience< MedlineTA>Neuroscience, 2003
